# Editorial: Computational systems immunovirology

**DOI:** 10.3389/fimmu.2023.1233547

**Published:** 2023-07-05

**Authors:** Mohadeseh Zarei Ghobadi, Majid Teymoori-Rad, Gurudeeban Selvaraj, Dong-Qing Wei

**Affiliations:** ^1^ Department of Virology, School of Public Health, Tehran University of Medical Sciences, Tehran, Iran; ^2^ Centre for Research in Molecular Modeling (CERMM), Department of Chemistry and Biochemistry, Concordia University, Montreal, QC, Canada; ^3^ College of Life Sciences and Biotechnology and State Key Lab. of Microbial Metabolism, Shanghai Jiao Tong University, Shanghai, China; ^4^ Zhongjing Research and Industrialization Institute of Chinese Medicine, Zhongguancun Scientific Park, Nanyang, Henan, China; ^5^ Peng Cheng Laboratory, Guangdong, China; ^6^ Centre for Research in Molecular Modeling, Concordia University, Montreal, QC, Canada

**Keywords:** systems immunovirology, viral vaccine, immunogenomics, deep learning analysis, mathematical modeling, machine learning model

Virus-immune system interplay has a significant effect on the final fate of infection. Therefore, it is essential to survey the pathogenesis mechanism of viral-caused disease through the alteration of the immune system function. One of the most effective approaches to surveying biological processes is systems biology, in which the whole system is studied rather than the individual parts (1, 2). Computational systems biology and machine/deep learning methods rely on mathematical and statistical algorithms as well as the modeling of biological systems (3, 4). These computational approaches can be employed in the analysis of immune-related data. This "Research Topic" highlights eight articles that have been published in “Frontiers in Immunology”. They aimed to utilize computational systems immunovirology for the following purposes: i) analysis of immunogenomics data for various aims; ii) developing a prediction model to find genome-scale protein-protein interactions between various virus strains and human proteome; iii) developing an online website to forecast the interplays among antigens and antibodies; iv) utilizing nonlinear ordinary differential equations to model the antibody dynamics; v) developing a multiscale mechanistic model for human Dendritic cells, vi) introducing a multilevel adapted prediction approach to detect the antiviral T cells produced by virotherapy; and viii) introducing a deep learning method to describe the gathered effects of multiple vaccine design variables.

Tumor growth can be impressed by viruses through particular target genes. Liao et al. evaluate the communication between the Severe Acute Respiratory Syndrome Coronavirus 2 (SARS-CoV-2) and malignancies. This was based on the idea that the immune system might be amplified by RNA vaccines of SARS-CoV-2 to destroy cancer (5). The authors assess the methylation, genomic mutation, clinical characteristics, immunological characteristics of SARS-CoV-2 target Genes (STGs), and activation of signature-related pathways in solid tumors. Furthermore, they construct risk prognostic models that rely on STGs, and their communications with immunology were identified among 33 types of tumors.

Zarei Ghobadi et al. survey the immunological molecular operators and other dysregulated events involved in the progression of COVID-19. They analyze coding and non-coding RNA-seq data, considering severe and non-severe conditions of disease, to propose a diagnostic immunological panel. They propose a co-expressed-based machine learning algorithm in which differential expression and weighted-gene co-expression analyses are utilized as the filter phase and a recursive feature elimination-support vector machine is performed as the wrapper phase. The results disclose two gene groups comprising 84 and 5 genes implicated in innate immune suppression and cell dysregulation, respectively. These genes classified patient groups from healthy subjects. Moreover, the role of demodulator miRNAs and their target genes contained in the metabolic pathways are identified. These molecular factors can determine the ultimate destiny of infection toward non-severe or severe COVID-19.

Protein-protein interactions (PPIs) between pathogen and host have important functions in the investigation of the immune response and infection mechanism. Kataria et al. develop a homology-based interolog and domain-based prediction model to figure out genome-scale PPIs between human proteome and 22 MPXV strains in the human-monkeypox virus pathosystem. The majority of the identified MPXV proteins constitute hubs with the AGC kinase C-terminal and protein kinases domains. Moreover, subcellular localization discloses the localization of large numbers of human proteins in the nucleus (26.79%) and cytoplasm (29.22%).

Humoral immunity is an indispensable sector of the immune system. The specific binding of antibodies to antigens could result in designing proper treatments for diseases. Huang et al. introduce an online tool named as AbAgIntPre, to forecast the interplays among antigens and antibodies based on the sequence traits. This tool gathers the composition of convolutional deep neural network framework and k-spaced amino acid pairs encoding for effectual forecasting of interactions. AbAgIntPre is available as a web server.

Besbassi et al. employ a nonlinear mixed modeling method to explore the dynamics of the immune response after re-exposure to varicella-zoster virus (VZV) endogenous. They apply various systems of nonlinear ordinary differential equations (ODEs) to model the antibody dynamics of VZV. The authors demonstrate that the appearance of VZV reactivation results in stopping the expansion of long-lived and short-lived antibody-secreting cells.

Dendritic cells (DCs) are a kind of antigen-presenting cell (APC) and phagocyte which modulate inflammatory responses of the immune system. Therefore, they are suitable therapeutic targets to reverse immune disease disturbances. A multiscale mechanistic model of human DCs is introduced by Aghamiri et al. It contains the complex interaction of intracellular molecular signaling to intercellular cell-cell communications. This study helps to perform computational experiments on human DC for drug discovery, immunotherapies, and vaccine design (6).

Oncolytic virotherapy is an introduced treatment for cancer by which viruses replicate more favorably in malignant cells and kill them (7). Vijver et al. determine the antiviral T cells generated by vesicular stomatitis virus-glycoprotein (VSV-GP) virotherapy in C57BL/6J mice. To distinguish between antiviral and antitumor T cells in the tumor, the VSV-GP epitopes that mouse anti-viral T cells reacted with are determined by employing a multilevel adapted bioinformatics viral epitope prediction method that relied on common neoepitope detection tools. The outcomes indicate that MHC-I alleles H2-Db and H2-Kb render VSV-GP epitopes which also induce the activation of T-cells.

The evolution of highly mutable infectious disease pathogens (hm-IDPs) is much quicker than the human immune system such that they can overcome customary vaccination. Faris et al. employ deep reinforcement learning to direct an agent-based model (8) of affinity maturation to concentrate sampling on immunization protocols. The approach also results in the amelioration of the broadly neutralizing antibody (bnAb) titers or generated bnAbs fraction. They coarse-grain a high number of vaccine design variables and follow the related design space. Their study leads to a proposal of a method to formulate vaccines with maximum protective immune replies to hm-IDPs.

Overall, the present research studies indicate the significance of computational algorithms in the analysis of virology data, developing efficient tools, modeling viral-host interactions and virus dynamics, detecting special antiviral cells, and investigating the effects of various variables in vaccine design. We hope these articles help researchers in their subsequent research.

## Author contributions

On behalf of all authors, MZG wrote the article. The authorsapproved the submitted version.
